# Dual-Channel Switchable Metasurface Filters for Compact Spectral Imaging with Deep Compressive Reconstruction

**DOI:** 10.3390/nano13212854

**Published:** 2023-10-27

**Authors:** Chang Wang, Xinyu Liu, Yang Zhang, Yan Sun, Zeqing Yu, Zhenrong Zheng

**Affiliations:** 1 College of Optical Science and Engineering, Zhejiang University, Hangzhou 310027, China; changwang_optics@zju.edu.cn (C.W.); 11930054@zju.edu.cn (X.L.);; 2 Intelligent Optics & Photonics Research Center, Jiaxing Research Institute, Zhejiang University, Jiaxing 314000, China

**Keywords:** optical metasurface, hyperspectral imaging, deep learning

## Abstract

Spectral imaging technology, which aims to capture images across multiple spectral channels and create a spectral data cube, has been widely utilized in various fields. However, conventional spectral imaging systems face challenges, such as slow acquisition speed and large size. The rapid development of optical metasurfaces, capable of manipulating light fields versatilely and miniaturizing optical components into ultrathin planar devices, offers a promising solution for compact hyperspectral imaging (HSI). This study proposes a compact snapshot compressive spectral imaging (SCSI) system by leveraging the spectral modulations of metasurfaces with dual-channel switchable metasurface filters and employing a deep-learning-based reconstruction algorithm. To achieve compactness, the proposed system integrates dual-channel switchable metasurface filters using twisted nematic liquid crystals (TNLCs) and anisotropic titanium dioxide (TiO_2_) nanostructures. These thin metasurface filters are closely attached to the image sensor, resulting in a compact system. The TNLCs possess a broadband linear polarization conversion ability, enabling the rapid switching of the incidence polarization state between *x*-polarization and *y*-polarization by applying different voltages. This polarization conversion facilitates the generation of two groups of transmittance spectra for wavelength-encoding, providing richer information for spectral data cube reconstruction compared to that of other snapshot compressive spectral imaging techniques. In addition, instead of employing classic iterative compressive sensing (CS) algorithms, an end-to-end residual neural network (ResNet) is utilized to reconstruct the spectral data cube. This neural network leverages the 2-frame snapshot measurements of orthogonal polarization channels. The proposed hyperspectral imaging technology demonstrates superior reconstruction quality and speed compared to those of the traditional compressive hyperspectral image recovery methods. As a result, it is expected that this technology will have substantial implications in various domains, including but not limited to object detection, face recognition, food safety, biomedical imaging, agriculture surveillance, and so on.

## 1. Introduction

Spectral imaging technology aims to capture images with multiple spectral channels, forming a spectral data cube essential for the identification, analysis, and classification of objects predicated on their distinctive spectral attributes. Hyperspectral imaging (HSI) technology has found extensive applications in remote sensing [1], medical diagnostics [2], object detection and recognition [3], agriculture surveillance [4], and other fields. However, the conventional spectral imaging methods, such as spatial scanning and spectral scanning, suffer from limitations, such as large volume and slow acquisition speed. To address these challenges, researchers have explored snapshot spectral imaging (SSI) [5,6,7] and compressive spectral imaging (CSI) [8,9,10,11] technologies. On the one hand, SSI systems have significantly improved the spectral image acquisition process. However, the early SSI methods faced limitations in obtaining a large number of spectral channels and relied on bulky optical systems for light splitting. On the other hand, CSI methods leverage compressive sensing (CS) theory [12,13] and encoding devices to block or filter the input light field, enabling the reconstruction of hyperspectral images with much fewer measurements than the number that Nyquist sampling requires. Further, snapshot compressive imaging (SCI) [6], a technique that captures high-dimensional (HD, ≥3D) data using a two-dimensional (2D) detector in very few shots, has been employed in spectral imaging, known as snapshot compressive spectral imaging (SCSI). SCSI was initially introduced as a dual-disperser coded aperture snapshot spectral imaging (DD-CASSI) [14] system, integrating compressive sensing into hyperspectral imaging. Numerous adapted hyperspectral imaging systems based on CASSI have emerged, including single-disperser CASSI (SD-CASSI) [15], multi-frame CASSI [16], dual-camera CSI [17], color-coded aperture CSI [18], and spatial–spectral encoded CSI [19]. However, the bulkiness and complexity of CASSI systems result in non-linear dispersion that necessitates calibration, leading to the degradation of spatial information and suboptimal recovery outcomes. Additionally, the energy loss of light and intricate optical components within the large system volume make CASSI frameworks impractical for portable applications. To overcome these limitations, phase-coded spectral imaging [20] has been developed to improve light throughput and reduce system volume, while wavelength-coded methods [20] have been pursued to achieve accurate and fast RGB-to-spectra recovery. Wavelength-coded approaches utilize RGB or broadband optical filters that can be extended to multiple designed broadband filters for precise wavelength encoding. In terms of spectral data cube decoding, CSI reconstruction algorithms can be categorized into model-based methods and learning-based methods [21]. The traditional iterative reconstruction approaches utilize designed measurements of the encoding process and prior knowledge. Thereinto, CS optimization algorithms, such as the two-step iterative shrinkage/thresholding (TwIST) algorithm [22], and prior conditions, like total variation (TV) regularization [23], have been introduced. Additionally, methods like basis function fitting [24] and dictionary learning [25] have been developed. However, these classic iterative algorithms often require long computation times and prior knowledge, resulting in limited reconstruction quality and applicability in mobile systems with speed requirements. With the fast development of planar optical elements as well as deep-learning algorithms, the compactness, reconstruction speediness, and quality of SCSI systems could be further improved.

For one thing, metasurfaces have gained considerable attention in recent years due to their ability to manipulate the incident wavefront versatilely with subwavelength resolution, offering control over amplitude [26,27,28,29], phase [30], polarization [31,32,33,34,35], and spectrum [36,37]. In addition, they can effectively miniaturize optical elements into compact, planar, and ultrathin devices. Thus, metasurfaces are suitable to be applied in spectral imaging systems [38,39] by functioning as broadband filters with diversified transmission spectra and working as compact and cost-efficient wavelength-coded apertures [40,41]. Furthermore, tunable and multifunctional metasurfaces provide greater design freedoms [42,43,44,45,46] with liquid crystals (LCs) being widely used for implementing tunable metasurfaces due to their unique birefringence properties and mature control mechanisms [45,46,47,48,49,50,51,52]. These characteristics possess significant potential in the implementation of compact, efficient, and precise SCSI systems.

In addition, deep-learning algorithms have emerged as an alternative for learning spatial–spectral priors and spectral reconstruction. They offer faster and more accurate reconstruction compared to iterative approaches, thanks to the strong fitting ability of deep-learning models that alleviates the high computation costs. Consequently, various deep neural network architectures, such as autoencoders [53], convolutional neural networks [54,55,56,57,58], generative adversarial networks [59], transformers [60], and others, have been utilized for spectral reconstruction. Additionally, the entire reconstruction process can be substituted with a neural network, and end-to-end (E2E) reconstruction allows for the sending of measurements into a deep neural network that directly outputs the reconstruction results. Specifically, residual neural networks (ResNet) [61,62], composed of convolutional layers with skip connections, have achieved outstanding performance in computer vision tasks, such as image classification and object detection, making them suitable for spectral reconstruction purposes.

This study proposes a novel snapshot compressive hyperspectral imaging system called MD-SCSI, which is based on dual-channel switchable metasurface filters and a deep-learning-empowered compressive reconstruction algorithm. MD-SCSI incorporates two key innovations: an SCSI hardware encoder that utilizes dual-channel switchable metasurface filters and a deep compressive reconstruction algorithm that employs E2E convolutional neural networks based on the ResNet architecture. On the one hand, the dual-channel switchable metasurface filters are constructed by integrating twisted nematic liquid crystals (TNLCs) with all-dielectric metasurfaces composed of anisotropic titanium dioxide (TiO_2_) meta-atoms. These thin metasurface filters are tightly integrated onto the image sensor, resulting in a compact system design. The TNLCs possess a broad linear polarization conversion capability [52], allowing for the rapid switching of the incidence polarization state between *x*-polarization and *y*-polarization by applying different voltages. This capability allows for the metasurface filters to operate as broadband optical filter arrays that are sensitive to polarization, thereby offering two distinct sets of transmittance spectra for wavelength encoding. Consequently, they facilitate the generation of 2-frame snapshots that capture spectral information, which in turn benefits the reconstruction of spectral data cubes. On the other hand, an E2E ResNet, instead of the traditional iterative CS algorithms, is employed to achieve hyperspectral imaging reconstruction. The ResNet is trained on a synthetic dataset using the 2-frame snapshot measurements obtained from the orthogonal polarization channels of MD-SCSI. By conducting a comparative analysis of the HSI reconstruction outcomes achieved using MD-SCSI against alternative methods, including a classical DD-CASSI method using random coded apertures and iterative CS algorithms with dictionary-learning based recovery (CASSI-DBR), method replacing the algorithm of MD-SCSI with iterative CS algorithms with dictionary-learning-based recovery (Meta-DBR), DD-CASSI using deep neural networks for recovery (CASSI-Net), and reconstruction with either the *x*-linear or *y*-linear polarization output channel of MD-SCSI (*x*-pol or *y*-pol). The precision and speediness of MD-SCSI have shown that MD-SCSI is superior to other methods, according to the improved reconstruction quality as well as speed. Generally, this research presents several contributions outlined as follows:

1. MD-SCSI realizes transmission spectra control by use of metasurfaces, employing two multiplexing input channels that work for orthogonally linear polarized light that can be rapidly switched using TNLCs. Additionally, the arrangement of meta-atoms within the metasurface units is optimized for minimization of coherence.
2. MD-SCSI achieves a compact SCSI framework rather than using additional elements or strategies of spatial-multiplexing, ensuring high-quality reconstruction while maintaining spatial resolution.
3. MD-SCSI enables fast and accurate HSI reconstruction by leveraging an end-to-end ResNet that is specially optimized for the dual-channel switchable metasurface filters, characterized by simplicity, high performance, rapid convergence, and exceptional generalization.

## 2. Materials and Methods

**Figure 1:**
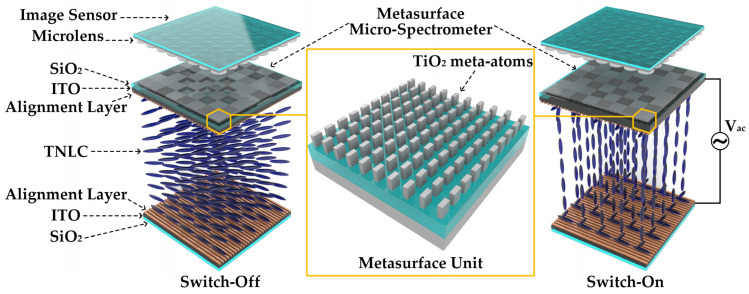
Schematic of the proposed MD-SCSI, consisting of a vertically stacked image sensor, a layer of dual-channel switchable metasurface filters with periodic micro-spectrometers, and a layer of TNLCs for tunable polarization conversion, enabling polarization channel selection of metasurface-filter arrays.

The proposed MD-SCSI system, as illustrated in Figure 1, consists of a vertically stacked image sensor, a layer of dual-channel switchable metasurface filters, and a layer of TNLCs. The metasurface layer is positioned between the sensor and the LC layer, and it comprises periodic micro-spectrometers. Each micro-spectrometer consists of 8 × 8 metasurface units with each unit measuring 3.45 μm × 3.45 μm, corresponding to a single complementary metal oxide semiconductor (CMOS) image sensor pixel. These metasurface units form a periodic array of 10 × 10 subwavelength anisotropic meta-atoms, which function as dual-channel switchable metasurface filters, exhibiting distinct transmission spectra for orthogonal polarization channels. The image sensor is integrated on top of the metasurface layer and accompanied by a micro lens array layer, while a thin TNLC cell is integrated beneath the metasurface layer to generate broadband linearly polarized incidences with orthogonal polarization states.

### 2.1. Metasurface Design

**Figure 2:**
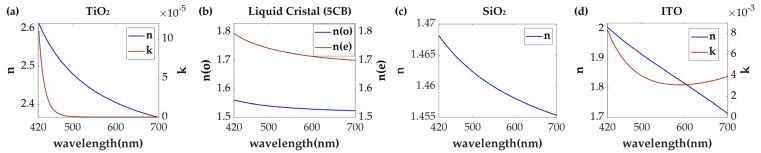
Refractive indices of (**a**) TiO_2_ [63], (**b**) TNLC [66], (**c**) SiO_2_ [64], and (**d**) ITO [67] materials used for constructing the meta-atoms.

**Figure 3:**
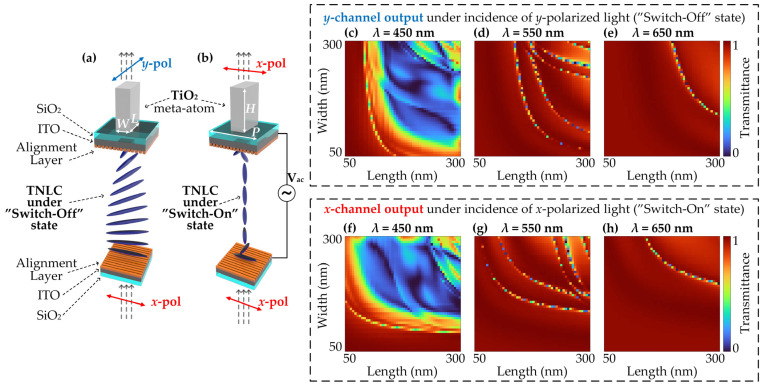
The detailed working principle of the proposed MD-SCSI. (**a**) The (helical) LC distribution in the ‘Switch-Off’ state under a meta-atom. (**b**) The situation in the ‘Switch-On’ state (no helical distribution) under a meta-atom. The polarization states of incident and output lights are indicated as red and blue arrows for *x*-polarization and *y*-polarization, respectively. (**c**–**e**) Diagram of transmittance response for different structural parameters of the meta-atoms under incidences of 450 nm, 550 nm, and 650 nm, respectively, when the LC distribution is in the ‘Switch-Off’ state and the *x*-polarized incidence is converted into *y*-polarization before hitting the meta-atom. (**f**–**h**) Diagram of transmittance response for different structural parameters of the meta-atoms under incidences of 450 nm, 550 nm, and 650 nm, respectively, when the LC distribution is in the ‘Switch-On’ state and the *x*-polarized incidence is maintained before hitting the meta-atom.

The device primarily consists of vertically stacked metasurface and TNLC layers. The upper metasurface layer is fabricated using high-ratio birefringent TiO_2_ meta-atoms [63] with varying cross-sectional shapes on top of a quartz (silicon dioxide, SiO_2_) substrate [64] (Figure 2). TiO_2_ naturally has an exceptionally low extinction coefficient (*k*), a large refractive index (*n*), and high transmittance in the visible range, making the energy of the light strongly confined within each meta-atom, and its negligible extinction coefficient across the visible spectrum avoids Ohmic loss. In addition, the proposed metasurface filters achieve dual-channel wavelength-coding by the anisotropic meta-atoms, which performs very diversified transmission spectra for *x*-pol or *y*-pol incidences with great design flexibility of its shape, size, arrangements, and so on. Further, this metasurface-based architecture implements an ultrathin and compact system with the potential of cost-effective mass production [65]. The underlying layer comprises a thin TNLC cell filled with the commonly used LC material 4-cyano-4′-pentylbiphenyl (5CB) [49,66], which includes two orthogonally oriented alignment layers to pre-align the LC molecules. The LC cell was sandwiched between two 10 nm-thick indium tin oxide (ITO) [67] layers. The proposed metasurface, integrated with TNLCs in the visible region, is demonstrated in Figure 3. By applying different voltages to the TNLC cell, the orientation of the LC molecules can be adjusted, enabling the conversion of linearly polarized light. In the absence of an electric field, vertically incident linearly polarized light with a polarization direction parallel to the first alignment layer undergoes a 90° deflection along the twisting direction of the LC molecules while passing through the TNLC cell. However, when a voltage that exceeds the threshold voltage is applied to the TNLC, the long axes of the LC molecules begin to incline in the direction of the electric field [48,52]. With the exception of the molecules near the alignment layers, the long axes of the remaining molecules tend to rearrange parallel to the electric field [48], leading to the suppression of LC molecule polarization conversion. Importantly, this optical rotation of the LC molecules is wavelength-independent, enabling the TNLC cell to operate over a broadband range.

The anisotropy characteristics and other optical behaviors in these meta-atoms can be described with the Jones matrix, denoted as ***J***_meta_ = [*t*_L_, 0; 0, *t*_S_], where *t*_L_ is the longer optical axis and *t*_S_ is the shorter optical axis. Thus, the complex transmittance ***E***_out_ from the meta-atom under an arbitrary incidence ***E***_in_ can be represented by

(1) $$E_{\mathrm{out}}=J_{\mathrm{meta}}E_{\mathrm{in}}.$$

For *x*-polarized incidence with ***E***_in_ = [*E_x_*_in_; *E_y_*_in_] = [1; 0], ***E***_out_ should be

(2) $$\left[\begin{array}{c} E_{x\mathrm{out}}\\ E_{y\mathrm{out}} \end{array}\right]=\left[\begin{array}{cc} t_{L} & 0\\ 0 & t_{S} \end{array}\right]\left[\begin{array}{c} 1\\ 0 \end{array}\right]=\left[\begin{array}{c} t_{L}\\ 0 \end{array}\right],$$

and for *y*-polarized incidence with ***E***_in_ = [*E_x_*_in_; *E_y_*_in_] = [0; 1], ***E***_out_ should be

(3) $$\left[\begin{array}{c} E_{x\mathrm{out}}\\ E_{y\mathrm{out}} \end{array}\right]=\left[\begin{array}{cc} t_{L} & 0\\ 0 & t_{S} \end{array}\right]\left[\begin{array}{c} 0\\ 1 \end{array}\right]=\left[\begin{array}{c} 0\\ t_{S} \end{array}\right].$$

The transmittance spectra of the proposed dual-channel meta-atoms were simulated using the finite difference time-domain (FDTD) method, specifically employing Lumerical FDTD Solutions. The meta-atoms had a period of 345 nm and a height of 600 nm. To account for fabrication constraints and period sizes, the meta-atoms were subjected to minimum and maximum size constraints of 50 nm and 300 nm, respectively. In order to facilitate the interpretation of the LC molecule behavior, the reorientation process of the LC was simulated accordingly. The equilibrium distribution of the LC director was calculated, and the resulting dielectric tensor field was incorporated into the FDTD simulations to determine the optical response. The computational domain was bounded above and below by a perfectly matched layer (PML) boundary condition with periodic conditions along the *x* and *y* directions.

A total of more than 2500 distinct meta-atoms were generated, out of which 64 types were carefully selected for spectral encoding purposes. These 64 structures were arranged in an 8 × 8 configuration, resembling a micro-spectrometer, and were subsequently replicated periodically to form a larger metasurface layer with a 256 × 256 array. When it comes to designing binary-coded apertures using only 0 and 1, an effective design rule that minimizes coherence involves the following considerations [68]: (1) maximizing the separation between one-valued entries within the same row of the coded aperture, (2) reducing the occurrence of vertical clusters in the vertical direction of the coded aperture, and (3) employing a complementary set of codes to minimize correlations, given that each measurement snapshot employs a distinct coded aperture. However, due to the meta-atoms’ ability to provide continuous transmittance control across the entire bandwidth, a slightly different yet similar strategy is adopted by employing a genetic algorithm. In both the horizontal and vertical directions, it is crucial to ensure a significant difference in transmittance between each pixel and its neighboring pixels. Furthermore, there should be substantial variation in transmittance for the meta-atoms within the same pixel when subjected to 2-frame snapshots of orthogonally polarized incidences. These requirements are essential to achieve the desired performance and characteristics of the metasurface.

### 2.2. Dual-Channel Compressive Hyperspectral Imaging with MD-SCSI

**Figure 4:**
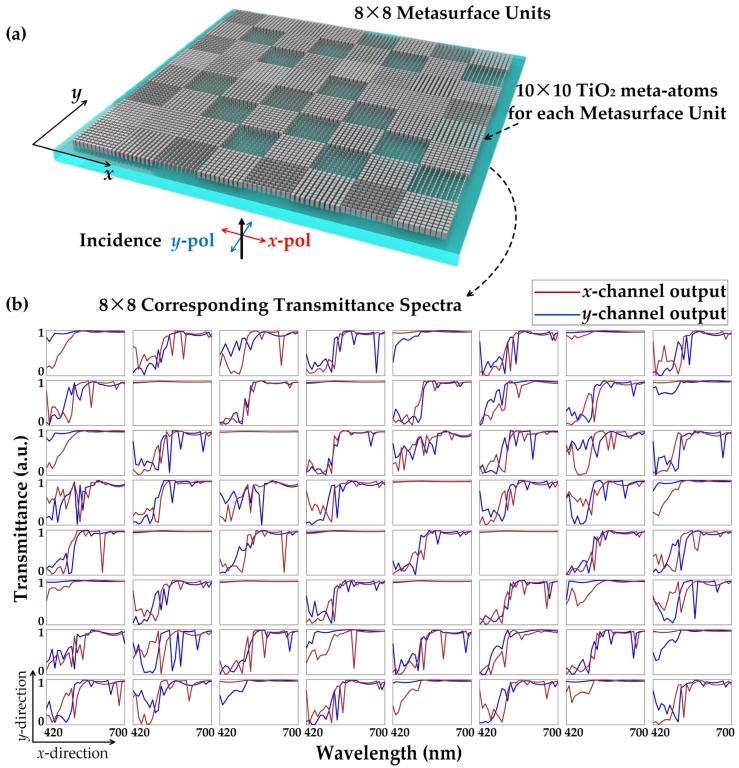
(**a**) View of the micro-spectrometer. Each micro-spectrometer consists of 8 × 8 metasurface units, and each metasurface unit is composed of 10 × 10 TiO_2_ meta-atoms. (**b**)Transmittance spectra of *x*-channel (red-line) and *y*-channel (blue-line) for the selected 8 × 8 metasurface units that make up the micro-spectrometer. The subfigures are arranged according to the relative location distributions of the 64 types of metasurface units along the *x*–*y* plane.

The spectral data cube of the incidence can be represented by *f*(*x*, *y*, *λ*), where (*x*, *y*) is the position on the metasurface plane and *λ* is the wavelength. Since every metasurface unit consists of 10 × 10 identical meta-atoms, its transmission spectra are the same as those of a single corresponding meta-atom. The transmission spectra tuning function can be denoted as *T_ix_*(*x*, *y*, *λ*) and *T_iy_*(*x*, *y*, *λ*) for *x*-polarization and *y*-polarization channels, respectively. Here, *i =* 1, 2, 3 … 64 represents the area number of the metasurface unit within the micro-spectrometer, as illustrated in Figure 4. Consequently, the pre-captured tuned data cube can be represented as *f*(*x*, *y*, *λ*)*T_ix_*(*x*, *y*, *λ*) and *f*(*x*, *y*, *λ*)*T_iy_*(*x*, *y*, *λ*). Furthermore, considering Ω(*λ*) as the spectral response function of the image sensor pixel, the finally detected measurement for pixel (*m*, *n*) can be expressed as follows:

(4) $$\left\{\begin{array}{l} g_{mnx}=\int_{(n-1)\Delta }^{n\Delta }\int_{(m-1)\Delta }^{m\Delta }\int_{\lambda }\Omega (\lambda )f(x,y,\lambda )T_{ix}(x,y,\lambda )d\lambda dxdy\\ g_{mny}=\int_{(n-1)\Delta }^{n\Delta }\int_{(m-1)\Delta }^{m\Delta }\int_{\lambda }\Omega (\lambda )f(x,y,\lambda )T_{iy}(x,y,\lambda )d\lambda dxdy \end{array}\right.,$$

where Δ = 3.45 μm is the period of one metasurface unit or one pixel of the image sensor, and (*m*, *n*) is the location of a particular pixel and corresponds to the metasurface unit number *i* from 64 types. If *λ* is a discrete parameter indicated by *s*, then the measurement of the pixel (*m*, *n*) would be

(5) $$\left\{\begin{array}{l} g_{mnx}=\sum_{s=1}^{N}\Omega_{s}f_{mns}T_{sx}^{i}\\ g_{mny}=\sum_{s=1}^{N}\Omega_{s}f_{mns}T_{sy}^{i} \end{array}\right.,$$

where *N* is the quantity of the spectral bands, and Ω*_s_* is the spectral response of the sensor for the *s*th spectral band. Supposing that the detected image has P rows and Q columns, add the total *g_mn_* and *f_mns_* to the detected 2D image **G** (**G** ∈ ℝ^2P×Q^) and the total 3D hyperspectral data cube **F** (**F** ∈ ℝ^P×Q×S^), respectively, and denote **g** (**g** ∈ ℝ^2PQ×1^) and **f** (**f** ∈ ℝ^PQS×1^) as the vectorization of **G** and **F**, respectively. Then the total detected signal can be expressed as

(6) g=Hf,

where **H** = [**H*_x_***; **H*_y_***] (**H** ∈ ℝ^2PQ × PQS^) is the observation matrix of MD-SCSI with dual polarization channels, as the number 2 in the superscripts of **G** and **H** denotes both channels. The total detected signal **g** can be represented by **g** = [**g*_x_***; **g*_y_***], where **g*_x_*** = **H*_x_*f** and **g*_y_*** = **H*_y_*f**.

### 2.3. Design of ResNet for Data Cube Reconstruction

**Figure 5:**
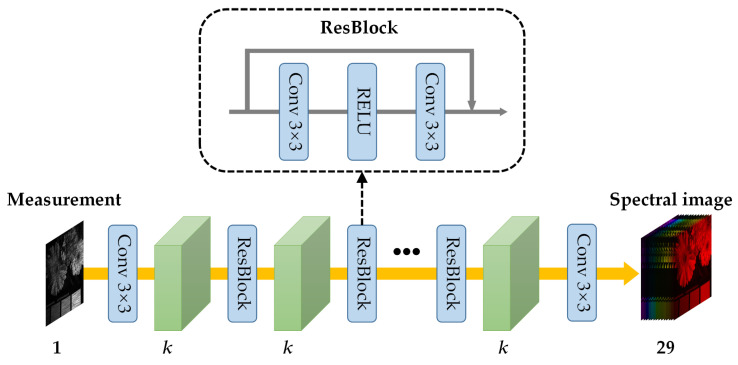
The framework of the utilized E2E ResNet for data cube reconstruction.

For data cube reconstruction, an end-to-end residual neural network (ResNet) is utilized. As a proof of principle, a subset of 29 wavelengths, ranging from 420 to 700 nm with a step size of 10 nm, is selected. The network architecture, depicted in Figure 5, initiates by applying a convolutional layer to transform the input measurements, which have a size of 256 × 256 × 1, into feature maps with dimensions 256 × 256 × K. Subsequently, N residual blocks are incorporated into the network. Finally, another convolutional layer is employed to convert the 256 × 256 × K feature maps into output channels with dimensions 256 × 256 × 29, corresponding to the selected wavelengths. Each residual block, as illustrated in the subfigure of Figure 5, comprises two convolutional layers and is enhanced with a rectified linear unit (ReLU) activation function. This composition enhances its ability to learn and capture the complex relationships within the data.

In this study, the CAVE dataset, comprising a collection of 32 hyperspectral images with dimensions of 512 × 512 × 32, is utilized for training purposes. Specifically, the parameters K and N are set to 72 and 20, respectively, to optimize the performance of the network. To enhance the training process, data augmentation techniques and spectral interpolation methods are employed, generating an extended dataset consisting of 272 hyperspectral images with dimensions of 1024 × 1024 × 29. For the purpose of testing, a set of 15 scenes obtained from KAIST is selected, each with dimensions of 256 × 256 × 29. These scenes provide a diverse range of real-world scenarios for evaluating the proposed approach. The computational resources employed in this study include an NVIDIA GTX 4090 GPU and an AMD 7950X CPU. These resources contribute to the efficient execution of the training and testing processes, enabling timely and accurate analysis of the hyperspectral data.

The formulated loss function encompasses three distinct components. The first component corresponds to the mean squared error (MSE) loss, quantifying the average squared discrepancy between the predicted values and the ground truth values:

(7) $$L_{\mathrm{mse}}={\left\|F_{pred}-F\right\|}_{2}^{2}.$$

The second component incorporates the structural similarity (SSIM) loss function, which assesses the structural similarity between the predicted values and the ground truth values by comparing the local patterns, luminance, and contrast of the predicted values with the true values. This loss function facilitates the evaluation of the perceptual quality of the reconstructed data:

(8) $$L_{\mathrm{SSIM}}=SSIM(F_{pred},F).$$

Lastly, the third component aims to minimize the spectral angle mapping (SAM). The SAM metric determines the similarity between the reconstructed spectra and the true spectra, focusing on the angular difference between them. The goal is to minimize this discrepancy, enhancing the spectral fidelity of the reconstructed data:

(9) $$\mathrm{SAM}=\sum_{i=1}^{256}\sum_{j=1}^{256}\cos^{-1}\left(\frac{\sum_{k=1}^{29}F_{pred}{}_{i,j,k}F_{i,j,k}}{{\left(\sum_{k=1}^{29}F_{pred}{{}_{i,j,k}}^{2}\right)}^{1/2}{\left(\sum_{k=1}^{29}F_{i,j,k}{}^{2}\right)}^{1/2}}\right).$$

Since there is no need to calculate cos^−1^ while training, to save time, the loss function of SAM is set as:

(10) $$L_{\mathrm{SAM}}=\sum_{i=1}^{256}\sum_{j=1}^{256}\left(\frac{\sum_{k=1}^{29}F_{pred}{}_{i,j,k}F_{i,j,k}}{{\left(\sum_{k=1}^{29}F_{pred}{{}_{i,j,k}}^{2}\right)}^{1/2}{\left(\sum_{k=1}^{29}F_{i,j,k}{}^{2}\right)}^{1/2}+\varepsilon }\right),$$

where *ε* = 1 × 10^−9^ represents a very small value to prevent division by zero. The overall loss function is the weighted sum of these three components:

(11) $$L=L_{\mathrm{mse}}+\alpha_{1}L_{\mathrm{SSIM}}-\alpha_{2}L_{\mathrm{SAM}},$$

where *α*_1_ = 0.05 and *α*_2_ = 0.01 are parameters to balance these three terms for this work.

## 3. Results

To assess the efficacy of the proposed method, a comprehensive evaluation is conducted utilizing three quantitative metrics to gauge the quality of the reconstructed hyperspectral images. These metrics encompass the SAM, peak signal-to-noise ratio (PSNR), and SSIM. The SAM metric serves as an indicator of spectral accuracy, ranging from 0 to 1. A smaller SAM value signifies a more precise representation of the spectrum. PSNR assesses the overall reconstruction quality, and higher PSNR values indicate superior reconstruction outcomes. SSIM measures the structural similarity between the restored images and the original ones with values ranging from 0 to 1. Larger SSIM values represent minimal distortion in the reconstructed images, implying a higher degree of similarity to the originals. To facilitate a fair and straightforward comparison, the selected test images are scaled from 0 to 1. This normalization simplifies the comparative analysis among the different methods. In the subsequent analysis, the proposed MD-SCSI method is benchmarked against various existing methods for CSI. These methods include CASSI-DBR, CASSI-Net, Meta-DBR, as well as *x*-pol or *y*-pol as introduced previously. By comparing the performance of the MD-SCSI method against these alternative approaches, a comprehensive evaluation of its effectiveness is conducted, illustrating its advantages and potential in terms of the quality and speed of spectral reconstructions.

**Figure 6:**
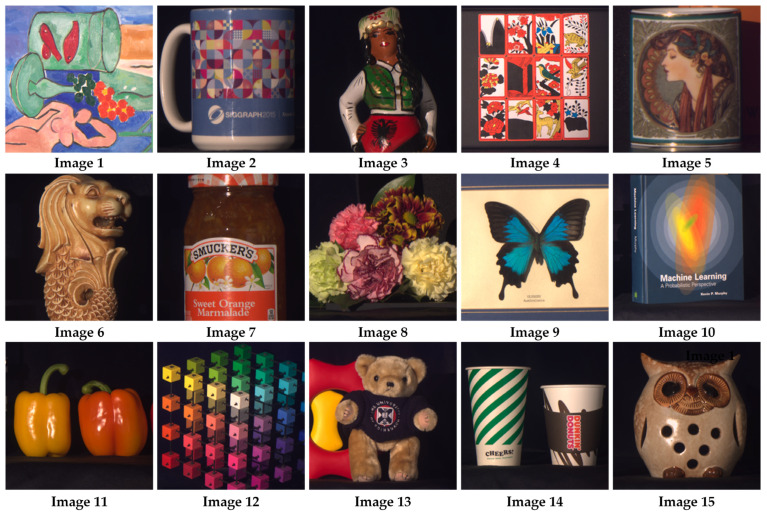
15 testing images from the KAIST dataset for simulation.

Table 1 showcases the reconstruction outcomes achieved through the application of diverse methodologies to a set of 15 images from the KAIST dataset. The findings firmly substantiate the exceptional reconstruction quality of the proposed MD-SCSI in comparison to the other methods. Across all 15 test images (Figure 6), the MD-SCSI method exhibits notable superiority in terms of SAM reduction, as well as significant enhancements in both PSNR and SSIM measures. These results underscore the remarkable efficacy of the proposed method, affirming its potential to outperform existing techniques in the domain of HSI reconstruction.

Moreover, an analysis of the comprehensive visual comparison results along with detailed information would provide further substantiation of the effectiveness of the proposed method. These illustrative demonstrations prove the improved performance and advantages offered by the proposed MD-SCSI when compared to alternative methods. By combining quantitative metrics with visual analysis, a compelling demonstration can be achieved, consistently showcasing the superior reconstruction quality achieved by the proposed method in comparison to other methods. This comprehensive evaluation, including both objective metrics and qualitative visual assessment, reinforces the efficacy of the proposed method and establishes its superiority in HSI reconstruction.

**Figure 7:**
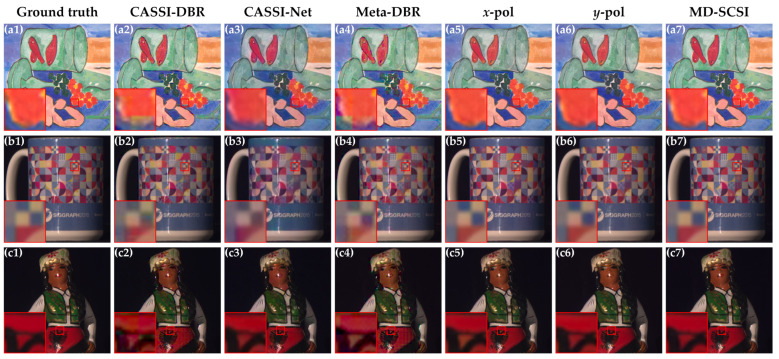
Visual comparison of reconstruction results using different methods with the 3D spectral data being synthesized into RGB images for intuitive display. (**a1**–**a7**) Ground truth compared with the results of CASSI-DBR, CASSI-Net, Meta-DBR, *x*-pol, *y*-pol, and MD-SCSI for image 1 in the KAIST dataset. (**b1**–**b7**) Ground truth compared with the results of CASSI-DBR, CASSI-Net, Meta-DBR, *x*-pol, *y*-pol, and MD-SCSI for image 2 in the KAIST dataset. (**c1**–**c7**) Ground truth compared with the results of CASSI-DBR, CASSI-Net, Meta-DBR, *x*-pol, *y*-pol, and MD-SCSI for image 3 in the KAIST dataset.

Figure 7 presents the reconstruction results of the different methods on three selected test images, and the reconstructed results produced with MD-SCSI (a7, b7, and c7) keep spatial details well with fine color fidelity, thereby demonstrating their superior reconstruction quality and precision. Conversely, it can be observed that CASSI-DBR exhibits significant noise, leading to noticeable blur, color distortion, and colored speckle noise as could be viewed in the zoomed-in versions. CASSI-Net and Meta-DBR achieve clearer reconstructed results; however, they still suffer from substantial color distortion or colored speckle noise overall. *x*-pol and *y*-pol produce satisfactory results with significantly improved clarity and colors that are closer to the ground truth. Nonetheless, some color distortion still persists, particularly in colors such as yellow and red. Finally, MD-SCSI yields the visually best results, characterized by clear outputs and colors that closely resemble the original images.

**Figure 8:**
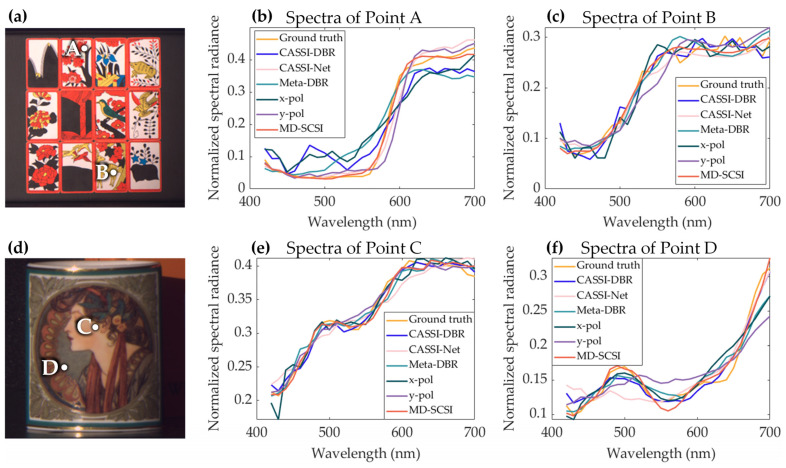
(**a**) Image 4 from the KAIST dataset with two randomly selected points, A and B, with the spectra of A and B that are reconstructed with different methods and depicted in (**b**,**c**), respectively. (**d**) Image 5 from the KAIST dataset with two randomly selected points, C and D, with the spectra of C and D that are reconstructed with different methods and depicted in (**e**,**f**), respectively.

In order to facilitate a more intuitive comparison in the spectral domain, Figure 8 illustrates the recovered spectra from two randomly chosen spatial positions in test images 4 and 5. It can be observed from Figure 8 that the spectral signatures reconstructed with MD-SCSI (orange lines) exhibit the best similarity to the ground truth spectra (yellow lines), and the average mean square errors (MSE) of the reconstructed spectra shown in Table 2 demonstrate that MD-SCSI is the most accurate statistically, further proving that MD-SCSI outperforms the other methods.

**Figure 9:**
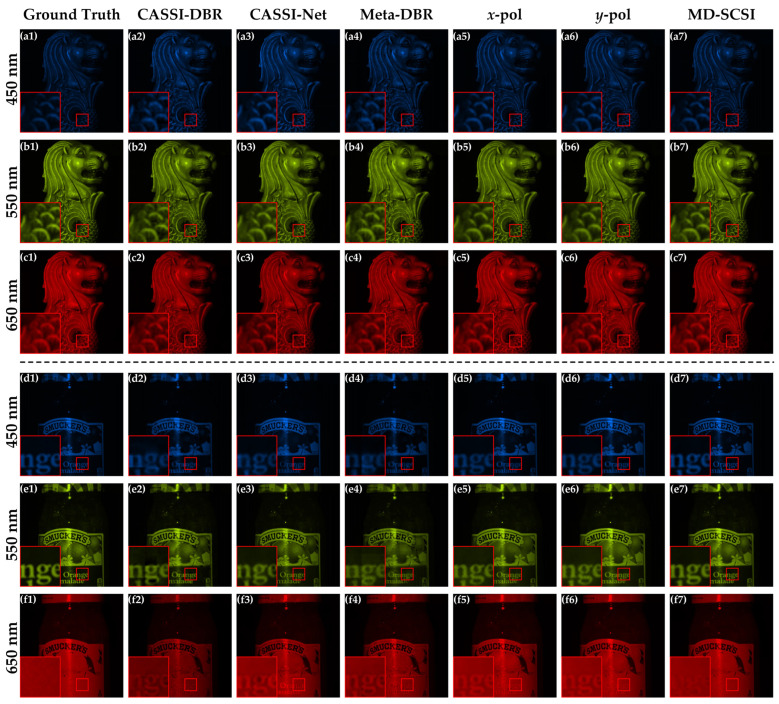
Visual comparison of image 6 for (**a1**–**a7**) 450 nm, (**b1**–**b7**) 550 nm, and (**c1**–**c7**) 650 nm with various methods compared to the corresponding ground truth, and visual comparison of image 7 for (**d1**–**d7**) 450 nm, (**e1**–**e7**) 550 nm, and (**f1**–**f7**) 650 nm with various methods compared to the corresponding ground truth.

Figure 9 displays the reconstruction results of two test images, image 6 and image 7, in three specific spectral channels of 450 nm, 550 nm, and 650 nm. It can be observed that the reconstruction results of CASSI-DBR, CASSI-Net, and Meta-DBR introduce obvious noise, resulting in relatively blurred details, shape deformations, and erroneous details in the 650 nm channel. The reconstruction results of *x*-pol and *y*-pol are closer to the ground truth; however, there are still incorrect details in the 650 nm band. Finally, MD-SCSI provides the most faithful representation of the ground truth, as it closely resembles the original images, exhibits fewer undesirable visual artifacts, and provides visually better reconstructions than the other methods without clear noises or erroneous details in the 650 nm band. This validates the superiority of MD-SCSI in this work.

**Figure 10:**
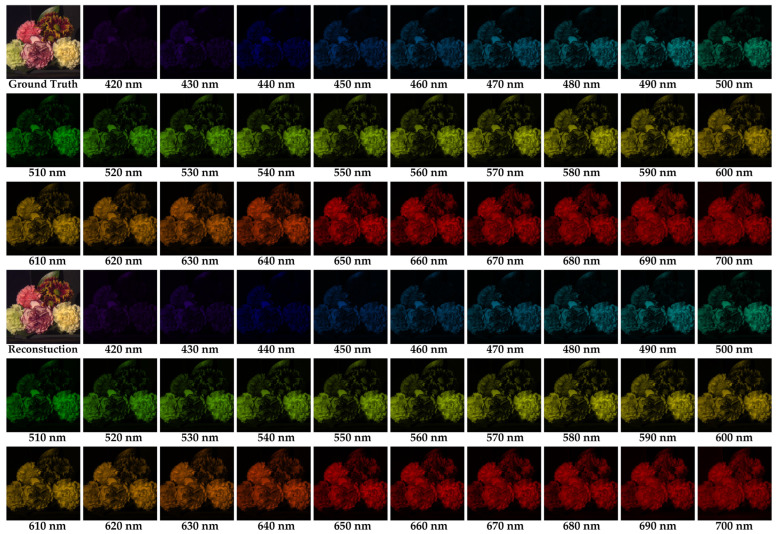
The ground truth of full-band spectra of image 8 with its reconstructed results of full-band spectra with the proposed MD-SCSI method.

Furthermore, Figure 10 showcases a comparison between the entire reconstructed hyperspectral image generated with MD-SCSI and the original image 8, and it clearly demonstrates that MD-SCSI generates reconstructed results that are visually pleasing and very close to the ground truths in terms of spatial structure, color fidelity, and edge details without noticeable noises or distortions.

Additionally, the average running time of MD-SCSI with the other contrasting methods on the 15 test images is listed in Table 3. Generally, the reconstructions of 15 test images are repeated 10 times for all six methods, including the proposed method, MD-SCSI, as well as the other contrasting methods. Thus, the running time for each method is the average of its corresponding 150 reconstruction times, respectively. Specifically, the PyTorch profiler tool is utilized for the evaluation of deep-learning-based methods (MD-SCSI, *x*-pol, *y*-pol, and CASSI-Net), and the timing function of MATLAB is adopted for the evaluation of the dictionary learning-based methods (CASSI-DBR and Meta-DBR). It could be noted that the methods based on neural networks require significantly less processing time, approximately in the order of 1 × 10^−3^, compared to the methods using iterative CS algorithms and dictionary learning, and the proposed E2E ResNet algorithm, which is optimized for MD-SCSI, performs the fastest reconstruction speed when similarly applied to *x*-pol, *y*-pol, and MD-SCSI. The deep-learning-based methods excel in rapid reconstruction, and this is because of the relatively straightforward matrix operations from parallel processing on GPUs as well as the inherent simplicity of the reconstruction process.

## 4. Conclusions

In conclusion, this paper proposes MD-SCSI, a compact snapshot hyperspectral compressive imaging system that leverages dual-channel switchable metasurface filters in conjunction with a deep-learning-empowered reconstruction algorithm based on compressive sensing theory. MD-SCSI presents a compact framework for snapshot hyperspectral imaging, eliminating the need for dispersion elements or additional cameras. This pioneering approach capitalizes on the unique advantages offered by dual-channel metasurfaces as SCSI hardware encoders while employing an end-to-end residual neural network for HSI reconstruction, thus demonstrating superiority in both system compactness and reconstruction performance. In detail, the dual-channel switchable metasurface filters are arranged according to minimization of coherence and integrated by TNLCs with broadband linear polarization conversion abilities. These metasurfaces enable rapid conversion between different voltages, generating 2-frame snapshots of spectral information with distinct transmittance spectra for each independent input channel, facilitating the reconstruction of spectral data cubes. Furthermore, the specially optimized end-to-end ResNet, tailored to the characteristics of the dual-channel metasurface filters, enables efficient HSI reconstruction by processing the 2-frame snapshot measurements acquired from orthogonal polarization channels of MD-SCSI. This reconstruction process achieves exceptional quality in terms of structural fidelity, color fidelity, spatial resolution, and speed. The comparison of the HSI reconstruction results obtained with MD-SCSI with the other approaches demonstrates the effectiveness, accuracy, as well as speediness of MD-SCSI, which greatly outperforms the other HSI reconstruction methods. Consequently, MD-SCSI exhibits tremendous potential for compact, accurate, and rapid SCSI and could be applied across various domains, including but not limited to food safety, biomedical imaging, precision agriculture, and object detection, among others.

## Figures and Tables

**Table 1:** SAM, PSNR, and SSIM comparison of different methods for 15 images in the KAIST dataset.

Image Number	Quality Metrics	CASSI-DBR	CASSI-Net	Meta-TwIST	*x*-pol	*y*-pol	MD-SCSI
Image 1	SAM	0.0860	0.1287	0.1095	0.0972	0.0989	**0.0570**
	PSNR	30.80	27.97	29.15	30.81	30.44	**35.59**
	SSIM	0.8867	0.8969	0.8713	0.9370	0.9357	**0.9643**
Image 2	SAM	0.0904	0.1364	0.1692	0.1220	0.1098	**0.0714**
	PSNR	35.03	31.65	32.92	35.51	35.61	**39.93**
	SSIM	0.9472	0.9393	0.9279	0.9739	0.9741	**0.9891**
Image 3	SAM	0.1332	0.1675	0.2689	0.1754	0.1604	**0.1106**
	PSNR	35.48	36.38	33.77	38.35	38.10	**41.84**
	SSIM	0.9533	0.9747	0.9275	0.9785	0.9808	**0.9914**
Image 4	SAM	0.1974	0.1314	0.2377	0.1139	0.1225	**0.0751**
	PSNR	30.28	31.57	28.94	33.01	31.96	**38.35**
	SSIM	0.8795	0.9415	0.8541	0.9533	0.9470	**0.9830**
Image 5	SAM	0.1036	0.1385	0.1324	0.1238	0.1266	**0.0765**
	PSNR	36.80	34.99	35.63	36.60	37.12	**40.95**
	SSIM	0.9557	0.9546	0.9508	0.9669	0.9672	**0.9845**
Image 6	SAM	0.0993	0.1105	0.1636	0.1078	0.1033	**0.0686**
	PSNR	35.17	34.44	33.65	36.20	35.55	**40.64**
	SSIM	0.9626	0.9728	0.9499	0.9820	0.9820	**0.9916**
Image 7	SAM	0.1001	0.1208	0.1595	0.1123	0.1065	**0.0692**
	PSNR	30.48	29.84	29.33	32.43	32.40	**36.89**
	SSIM	0.9166	0.9340	0.9005	0.9524	0.9527	**0.9738**
Image 8	SAM	0.1194	0.1378	0.1970	0.1476	0.1494	**0.0752**
	PSNR	35.14	32.21	32.73	32.58	32.58	**39.21**
	SSIM	0.9368	0.9470	0.9136	0.9570	0.9561	**0.9856**
Image 9	SAM	0.0799	0.0913	0.0946	0.0642	0.0700	**0.0449**
	PSNR	36.70	33.76	35.68	37.49	36.41	**39.78**
	SSIM	0.9388	0.9455	0.9402	0.9705	0.9661	**0.9781**
Image 10	SAM	0.0813	0.1081	0.1518	0.1016	0.0870	**0.0580**
	PSNR	42.37	40.26	39.45	41.41	42.09	**47.90**
	SSIM	0.9820	0.9827	0.9701	0.9894	0.9906	**0.9959**
Image 11	SAM	0.0967	0.1427	0.2434	0.1310	0.1248	**0.0893**
	PSNR	39.2121	37.70	37.12	40.57	40.76	**43.70**
	SSIM	0.9656	0.9743	0.9335	0.9841	0.9870	**0.9929**
Image 12	SAM	0.2171	0.1864	0.2920	0.1639	0.1557	**0.1037**
	PSNR	35.4471	36.3229	33.4164	39.5796	39.9063	**44.5338**
	SSIM	0.9060	0.9633	0.8706	0.9790	0.9800	**0.9917**
Image 13	SAM	0.1030	0.1208	0.1945	0.1202	0.1128	**0.0785**
	PSNR	38.1660	38.4325	35.0692	40.8228	40.5658	**44.1873**
	SSIM	0.9565	0.9777	0.9321	0.9855	0.9863	**0.9926**
Image 14	SAM	0.1071	0.1440	0.2363	0.1419	0.1304	**0.0878**
	PSNR	33.6037	33.3215	32.2650	36.1854	35.8398	**40.6773**
	SSIM	0.9641	0.9751	0.9454	0.9817	0.9832	**0.9921**
Image 15	SAM	0.0897	0.1172	0.1928	0.1253	0.1124	**0.0798**
	PSNR	38.8602	39.2061	37.3496	41.3150	41.3412	**44.2754**
	SSIM	0.9751	0.9821	0.9608	0.9861	0.9874	**0.9937**
Average	SAM	0.1136	0.1321	0.1896	0.1232	0.1180	**0.0764**
	PSNR	35.5685	34.5362	33.7651	36.8566	36.7112	**41.2303**
	SSIM	0.9418	0.9574	0.9232	0.9718	0.9718	**0.9867**

**Table 2:** Average mean square error of the spectra for the selected points depicted in Figure 8.

Image	CASSI-DBR	CASSI-Net	Meta-DBR	*x*-pol	*y*-pol	MD-SCSI
Image 4	1.771 × 10^−4^	1.800 × 10^−4^	3.565 × 10^−4^	1.565 × 10^−4^	2.425 × 10^−4^	**9.184 × 10^−5^**
Image 5	9.310 × 10^−5^	1.109 × 10^−4^	1.645 × 10^−4^	1.671 × 10^−4^	1.456 × 10^−4^	**3.119 × 10^−5^**

**Table 3:** Average running time on the test dataset for different methods.

Method	CASSI-DBR	CASSI-Net	Meta-DBR	*x*-pol	*y*-pol	MD-SCSI
Running Time	138.1021 s	118.3007 ms	135.5728 s	115.3872 ms	116.9829 ms	115.9519 ms

## Data Availability

The data underlying the results presented in this paper are not publicly available at this time but may be obtained from the authors upon reasonable request.
